# Effects of constant temperature and daily fluctuating temperature on the transovarial transmission and life cycle of *Aedes albopictus* infected with Zika virus

**DOI:** 10.3389/fmicb.2022.1075362

**Published:** 2023-01-04

**Authors:** Xian-yi Jian, Yu-ting Jiang, Miao Wang, Nan Jia, Tong Cai, Dan Xing, Chun-xiao Li, Tong-yan Zhao, Xiao-xia Guo, Jia-hong Wu

**Affiliations:** ^1^The Key and Characteristic Laboratory of Modern Pathogen Biology, School of Basic Medical Sciences, Guizhou Medical University, Guiyang, China; ^2^State Key Laboratory of Pathogens and Biosecurity, Beijing Institute of Microbiology and Epidemiology, Beijing, China

**Keywords:** *Aedes albopictus*, Zika virus, transmission efficacy, life cycle, daily fluctuating temperature

## Abstract

**Introduction:**

Numerous studies on the mosquito life cycle and transmission efficacy were performed under constant temperatures. Mosquito in wild, however, is not exposed to constant temperature but is faced with temperature variation on a daily basis.

**Methods:**

In the present study, the mosquito life cycle and Zika virus transmission efficiency were conducted at daily fluctuating temperatures and constant temperatures. *Aedes albopictus* was infected with the Zika virus orally. The oviposition and survival of the infected mosquitoes and hatching rate, the growth cycle of larvae at each stage, and the infection rate (IR) of the progeny mosquitoes were performed at two constant temperatures (23°C and 31°C) and a daily temperature range (DTR, 23–31°C).

**Results:**

It showed that the biological parameters of mosquitoes under DTR conditions were significantly different from that under constant temperatures. Mosquitoes in DTR survived longer, laid more eggs (mean number: 36.5 vs. 24.2), and had a higher hatching rate (72.3% vs. 46.5%) but a lower pupation rate (37.9% vs. 81.1%) and emergence rate (72.7% vs. 91.7%) than that in the high-temperature group (constant 31°C). When compared to the low-temperature group (constant 23°C), larvae mosquitoes in DTR developed faster (median days: 9 vs. 23.5) and adult mosquitoes carried higher Zika viral RNA load (median log_10_ RNA copies/μl: 5.28 vs. 3.86). However, the temperature or temperature pattern has no effect on transovarial transmission.

**Discussion:**

Those results indicated that there are significant differences between mosquito development and reproductive cycles under fluctuating and constant temperature conditions, and fluctuating temperature is more favorable for mosquitos' survival and reproduction. The data would support mapping and predicting the distribution of *Aedes* mosquitoes in the future and establishing an early warning system for Zika virus epidemics.

## 1. Introduction

*Aedes albopictus*, which is widely distributed in China, is an important transmission vector for many important mosquito-borne infectious diseases such as Zika virus diseases and dengue fever. In recent years, urbanization and population migration have caused global climate change, and the distribution of *Ae. albopictus* has become widespread in China. It is predicted that in the 2050s, the expansion of *Ae. albopictus* will mainly occur in central and northern areas of China and most of southern China will become a habitat for *Ae. albopictus* (Liu et al., 2019).

The temperature has a major impact on the biological characteristics of mosquitoes (mosquito survival rate, fecundity, etc.) and the interaction between mosquitoes and mosquito-borne viruses. *Ae. albopictus* fertility and temperature are positively correlated, and the number of eggs laid increases with the increase in temperature (Carrington et al., 2013a), but the raising temperature will also increase the mortality rate of the adults. The survival rate of *Ae. albopictus* is the highest at 15°C and the lowest at 35°C (Delatte et al., 2009). However, the effect of temperature on fertility and survival under virus infection has not been reported. Transovarial transmission is the spread of a pathogen from parental to progeny, and infection of the parental mosquitoes directly affects transovarial transmission capacity. Currently, laboratory studies about the effect of temperature on biological characteristics and infection of mosquitoes with viruses were performed under constant temperature, and constant temperature affected infection rate (IR) in a unimodal way (Liu-Helmersson et al., 2014; Tesla et al., 2018), which exhibited the highest IR at a range of optimum temperature (28–34°C) and IR decreased in the extremely high and low temperatures.

Mosquitoes in wild, however, are not exposed to constant temperature but are faced with temperature variation on a daily basis, and those two different patterns might influence the biological parameters of mosquitoes differently. For example, significant differences in survival rates were observed between laboratory and field experiments (Brady et al., 2013). Nayar reported that the life span of *Aedes* was temperature-dependent at constant temperatures, but this rule deviated at fluctuating temperatures (Nayar, 1972). Joshi compared the development, adult longevity, and fecundity of *Aedes krombeini* between a series of fluctuating temperatures and their mean constant temperatures and found that longevity time and fecundity values were statistically different between the two conditions, but development times were different only in males (Joshi, 1996). A recent study reported that the development time of *Aedes aegypti* increased but fecundity reduced at both low (16°C) and high (35°C) daily fluctuating temperatures relative to the mean constant temperature (Carrington et al., 2013a).

Therefore, we assume that fluctuating temperatures have different impacts on mosquito development and virus invasion and propagation in mosquitoes compared to a constant temperature. Here, we investigated the effects of different temperature patterns on mosquito biological parameters (e.g., development, survival, adult longevity, and fecundity) and transovarial transmission and provided data to support the distribution and control of *Ae. albopictus* populations and early warning of Zika virus epidemics.

## 2. Materials and methods

### 2.1. *Ae. albopictus* colonies

*Ae. albopictus* was collected from Guangzhou (geographical coordinates: N23°07′, E113°16′) and cultivated up to the 20th generation in the Beijing Institute of Microbiology and Epidemiology. Mosquitoes were reared at 25 ± 1°C and 75 ± 5% relative humidity (RH), under a 14:10 light/dark (L/D) photoperiod, and adult mosquitoes were fed a 10% glucose solution daily.

### 2.2. Virus

Zika virus SZ01 strain (GenBank: KU866423) was deposited in the Microbial Culture Collection Center of the Beijing Institute of Microbiology and Epidemiology. The virus was isolated from the blood of a Chinese returning from Samoa in 2016 and has been passaged six times in C6/36 cells. The virus titer used in this study was 1.24 × 10^7^ plaque-forming units per ml (PFU/ml), and the virus was stored at −80°C.

### 2.3. Temperature design

**Figure 1:**
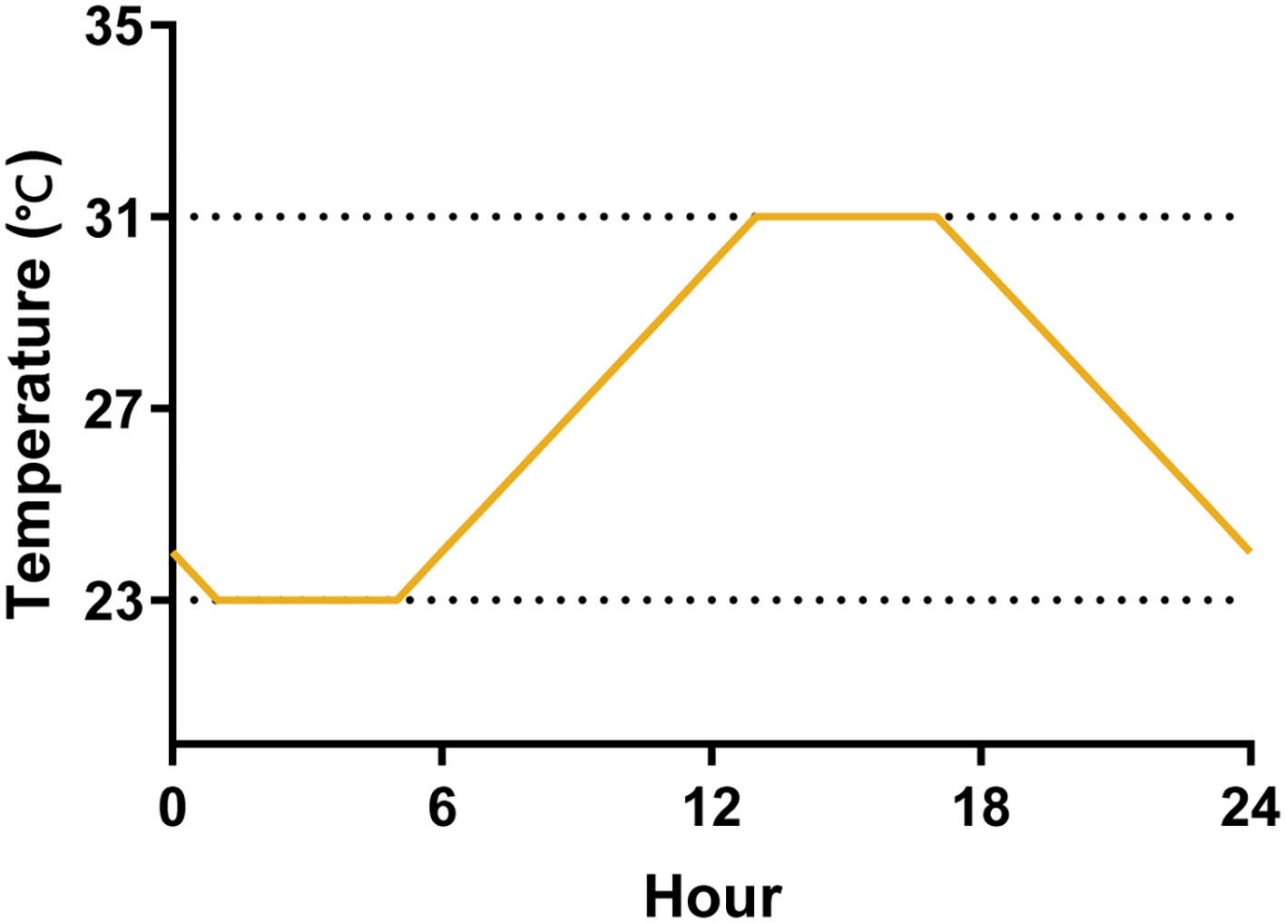
A diagram of the temperature fluctuations of DTR for 1 day. The temperature was maintained at 23°C from 1:00 to 5:00, and gradually increased from 23 to 31°C from 5:00 to 13:00, then it was maintained at 31°C until 17:00, and finally dropped to 23°C from 17:00 to 1:00.

We evaluated the effect of temperature variations on the life cycle and transmission efficacy of *Ae. albopictus* experimentally using three temperature regimes by programmable incubators (Shanghai Yiheng, MGC-350BP-2). The constant temperature has two groups, 23°C and 31°C, and they are the mean minimum and maximum temperatures of seasons with high mosquito-borne virus epidemics in Guangdong Province, China, in the recent 5 years (Cai et al., 2020). The daily temperature range (DTR) fluctuated between the maximum and minimum temperatures according to the daily temperature fluctuation pattern, reaching the minimum temperature at 1:00 and the maximum temperature at 13:00 (Figure 1). We programmed a photoperiod of 14:10 (L:D) h cycle, with alternations occurring at 6:00 a.m. and 8:00 p.m. and RH maintained at 75 ± 5% across all experiments. We monitored RH, air, and water temperature using a hygrometer.

### 2.4. Oral infection of mosquitoes

After emergence, about 2,500 female mosquitoes that were 4–6 days old were deprived of sucrose solution for 24 h before being offered the infectious blood meal. The blood meal is prepared by mixing the Zika virus suspension with fresh mouse blood in a ratio of 1:1. The virus blood meal was retained at 37°C by a multifunctional insect blood supply device (Hemotek, PS6A Power Unit) and supplied to the mosquitoes for 1 h. Fully engorged females were then transferred to a single culture tube and maintained at two constant temperatures of 23 and 31°C and a DTR of 23–31°C, respectively, until 4 days post-infection (dpi), when oviposition was finished.

### 2.5. Transovarial transmission

The female mosquitoes from three temperature groups that have completed egg laying in the culture tube are anesthetized with CO_2_, from which the total RNA is extracted to determine Zika virus infection. The egg paper of the virus-positive female mosquitoes was held in desiccators at 25 ± 1°C and 90% RH with a daily photoperiod of 14:10 L:D for 6 days to permit embryonation. The egg paper was then placed at the corresponding temperature for hatching, and when the offspring mosquitoes emerged, they were anesthetized. The whole mosquito RNA was extracted, and qRT-PCR was used to detect whether each progeny mosquito carried the Zika virus.

### 2.6. Virus detection

The virus was quantified by qRT-PCR using the GoTaq^®^ Probe 1-Step RT-qPCR System (Promega A6120). The PCR primers included a pair of universal primers and probe (Pyke et al., 2014), F: 5'-AAGTTTGCATGCTCCAAGAAAAT-3', R: 5'-CAGCATTATCCGGTACTCCA-3', and probe: 5' FAM-ACCGGGAAGAGCATCCAGCCAGA-3' BHQ1. The reaction condition is 45°C for 15 min; 95°C for 10 min; and 40 cycles of 95°C for 15 s, and 60°C for 60 s.

### 2.7. Life cycle

Three groups, i.e., 23, 31, and 23–31°C with DTR conditions, were set in the experiment. Each group contains 50 fully engorged females that were placed in a feeding tube individually. Filter paper placed in a small container with water was supplied to the mosquitoes for oviposition 4 days after the blood meal. The eggs laid by each female mosquito were counted 4 days after the filter paper was placed, and then held in desiccators at 25 ± 1°C and 90% RH with a daily photoperiod of 14:10 L:D for 6 days to permit embryonation. The developed eggs were incubated in three different temperature conditions described previously to hatch. The total number of larvae, pupae, and adult mosquitoes in each group was counted. The larvae development time was defined as the time from the second day of hatching to the day of pupation. The hatching rate, pupation rate, and emergence rate were calculated as follows:


$$\begin{array}{l} \text{ Hatching rate }=\text{ number of larvae}/\text{number of eggs }\\ \text{ Pupation rate }=\text{ number of pupae}/\text{number of larvae }\\ \text{Emergence rate }=\text{ number of adult mosquitoes}/\text{number }\\ \;of\;pupae \end{array}$$

### 2.8. Statistical analysis

The data analysis was performed using the Graphpad Prism 8.02 software, and the normality of residuals was tested by the Anderson–Darling (A2^*^) test. The survival curve of each group was compared by the log-rank (Mantel–Cox) test with Bonferroni correction. The number of mosquito eggs was analyzed by one-way ANOVA with Holm–Sidak's multiple comparisons test. The life cycle (hatching rate, pupation rate, and emergence rate) and IR of parental and progeny were analyzed by the chi-square test. Kruskal–Wallis test with Dunn's multiple comparisons test was used to analyze the development time and Zika virus viral load of the mosquitoes.

## 3. Results

### 3.1. The effect of temperature on mosquito survival and reproduction after Zika virus infection

**Figure 2:**
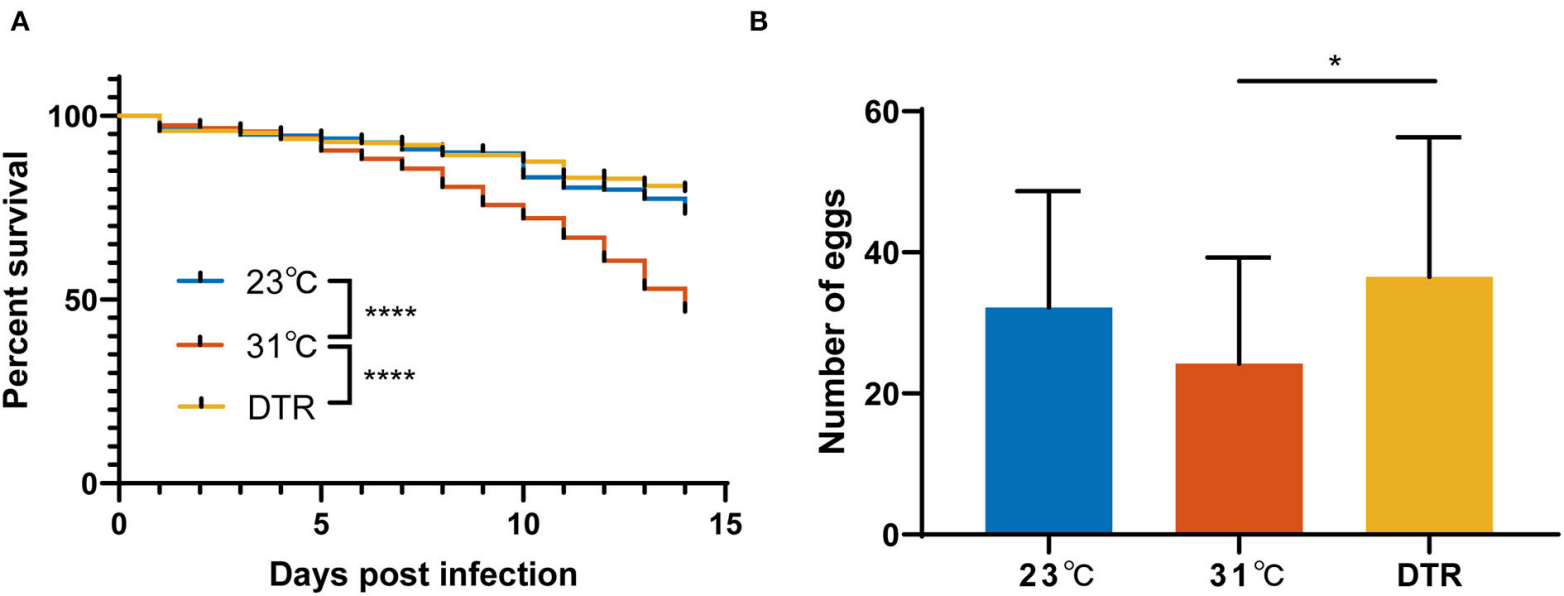
Survival curves and reproduction of *Ae. albopictus* infected with Zika virus at different temperatures. **(A)** Mortality of *Ae. albopictus* was monitored and recorded daily after the Zika virus infection. The survival curve of each group was compared by log-rank (Mantel–Cox) test with Bonferroni correction. *N* = 400–500. **(B)** The number of eggs laid by *Ae. albopictus* mosquitoes after Zika virus infection. Data were presented as mean ± SD. The number of eggs was analyzed by one-way ANOVA with Holm–Sidak's multiple comparisons test. *N* = 23–43. *P < 0.05, ****P < 0.0001.

To evaluate the effect of different living conditions on mosquitoes, the survival curve of *Ae. albopictus*, which were reared under constant temperatures at 23 and 31°C or DTR condition after Zika virus infection, was monitored. The survival rate of *Ae. albopictus* is the lowest at 31°C and significantly lower than the other two groups (log-rank, *P* < 0.0001; Figure 2). The survival rate of *Ae. albopictus* in the DTR group is highest, but there was no significant difference between the 23°C and DTR groups (log-rank, *P* = 0.0665). The total number of eggs that each mosquito laid was also counted to evaluate the effect of temperature on mosquito reproduction. Results showed that mosquitoes in the DTR group laid more eggs than that in 31°C (36.5 ± 19.8 vs. 24.2 ± 15.1). These results indicated that fluctuating temperature was more beneficial for mosquitoes' survival and maintenance.

### 3.2. The effects of constant temperature and daily fluctuating temperature on the mosquito life cycle

**Figure 3:**
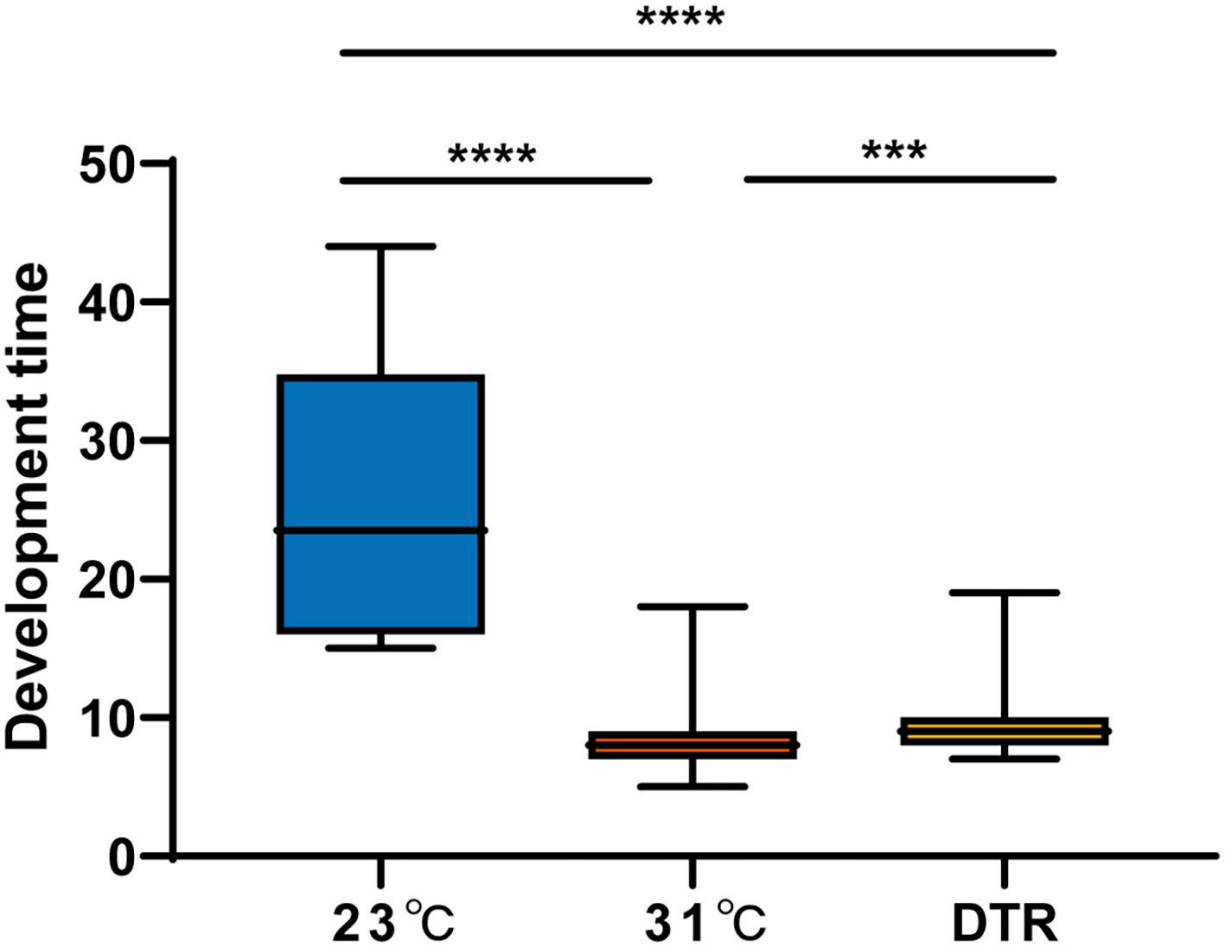
The development time of *Ae. albopictus* (F1) at different temperatures. The larvae development time was defined as the time from the second day of hatching to the day of pupation. Box plots show the median and the 25–75th percentiles, and the whiskers denote the maximum and minimum values. Data were analyzed by the Kruskal–Wallis test with Dunn's multiple comparisons test. *N* = 1,108–259. ***P < 0.001, ****P < 0.0001.

The development time was the longest in the low-temperature group at 23°C, with a median of 23.5 days, which was significantly longer than the other two groups (Figure 3). The high-temperature group had the lowest hatching rate (46.5%), which was significantly lower than the other two groups (*P* < 0.001) but had the highest pupation rate (significantly higher than the DTR group, *P* < 0.0001), emergence rate (significantly higher than the other two groups, *P* < 0.01, Table 1), and the shortest development time (median: 8 days). The DTR group exhibited the opposite profile of the high-temperature group, with the highest hatching rate and the lowest pupation rate (significantly lower than the other two groups) and emergence rate.

**Table 1:** Effects of temperature on mosquitoes' life cycle.

**Temperature**	**Hatching rate (%)**	**Pupation rate (%)**	**Emergence rate (%)**
23°C	67.2a^a^	63.5a	78.4a
31°C	46.5b	81.1b	91.7b
DTR	72.3a	37.9a	72.7a

^a^Values in a column followed by the same letter are not significantly different (the hatching rate, pupation rate, and emergence rate of mosquitoes were analyzed by chi-square test, P < 0.05). A total of 1,222, 557, and 1,533 eggs were used in the group of 23°C, 31°C, and DTR, respectively.

### 3.3. The effect of temperature on transovarial transmission

For a more comprehensive description of the effect of temperature on the transmission efficacy of the Zika virus by *Ae. albopictus*, Zika virus IR and viral RNA load in the parental females (F0) and the progeny (F1) from virus-positive F0 females were tested.

**Figure 4:**
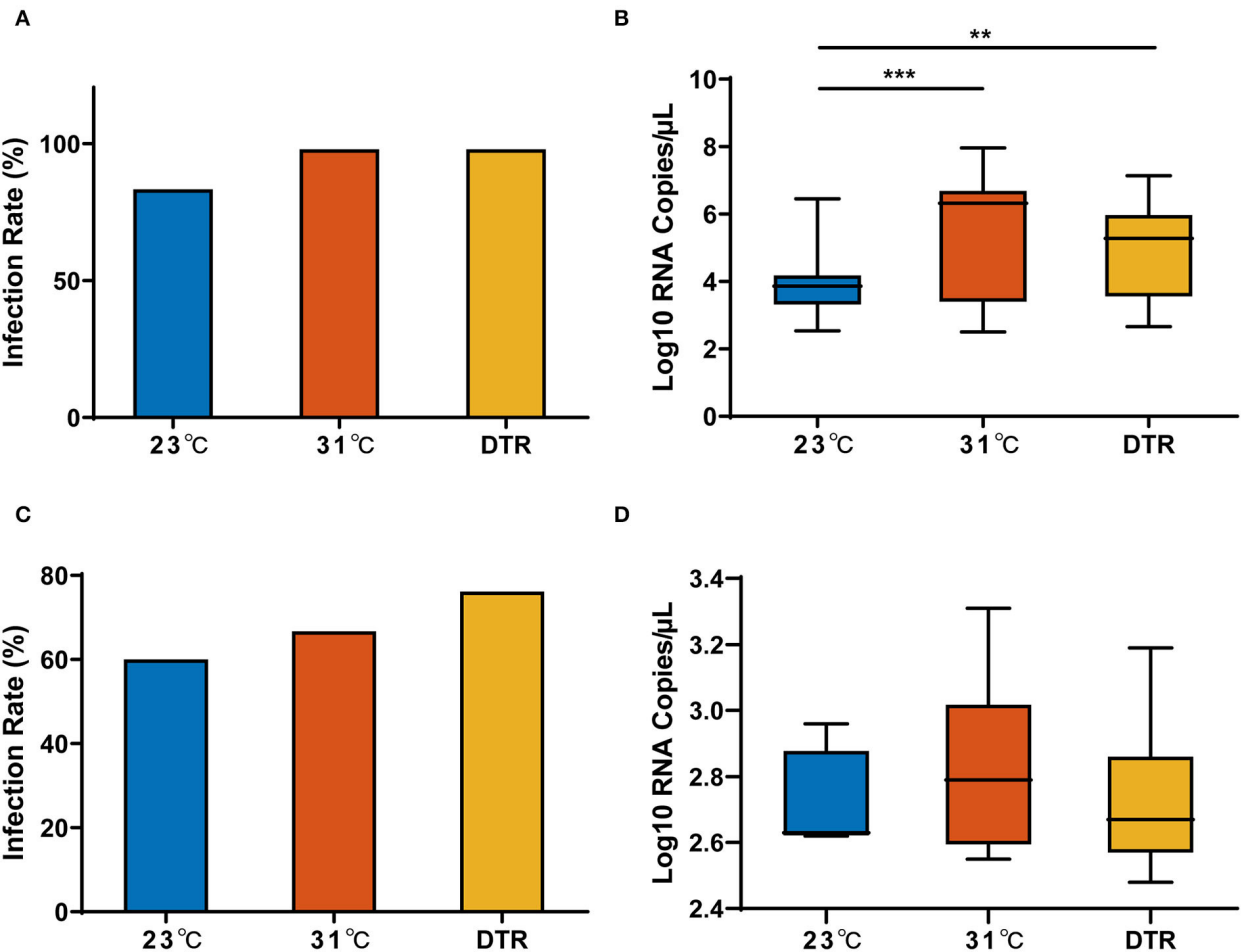
Zika virus infection rate and load in parental and progeny mosquitoes at different temperatures. Zika virus infection rate **(A)** and RNA copy number **(B)** in the parental *Ae. albopictus*; Zika virus infection rate **(C)** and RNA copy number **(D)** in the progeny *Ae. albopictus*. IR of parental mosquitoes, n = 50; IR of progeny mosquitoes, n = 6–21. Box plots show the median and the 25–75th percentiles, and the whiskers denote the maximum and minimum values. The chi-square test was used to analyze the IR and Kruskal–Wallis test with Dunn's multiple comparisons test was used to analyze the Zika virus viral load of the mosquitoes. **P < 0.01, ***P < 0.001.

The IR and the Zika virus RNA copy in F0 females from three temperature groups (23, 31, and 23–31°C DTR) are presented in Figure 4. For the IR of F0 females, there was no significant difference in the three temperature groups. However, the Zika virus RNA copy in the three groups was significantly different. The lowest load was detected at 23°C with a median of 3.86 log_10_ RNA copies/μl, and it was significantly lower than that in 31°C (median: 6.32 log_10_ RNA copies/μl) and DTR (median: 5.28 log_10_ RNA copies/μl) groups. Thus, temperature had no effect on virus invasion to the midgut but had a significant effect on virus replication and proliferation. Interestingly, IR and Zika virus load in F1 female mosquitoes were similar between the three temperatures.

## 4. Discussion

The life cycle of a mosquito is highly susceptible to the temperature, including the survival rate of the adults, life span, fecundity, and transovarial transmission ability. The present study showed that there were significant differences in the survival rate, hatching rate, and virus IR in the progeny of *Ae. albopictus* infected with the Zika virus under a DTR compared with a constant temperature.

### 4.1. Survival, fecundity, and transovarial transmission of adult mosquito

The results showed that, in the constant temperature condition, the survival rate of *Ae. albopictus* in the high-temperature group was lower than that in the low-temperature group, which is similar to the results of other studies on the survival rate of *Aedes aegypti* (Delatte et al., 2009; Onyango et al., 2020). Although the survival rate differs among mosquito species, the trend of temperature affecting survival rate similar to the present study was still found in *Culex* and *Anopheles* mosquitoes (Aytekin et al., 2009; Faiman et al., 2017; Barreaux et al., 2018; Spanoudis et al., 2019). Spanoudis et al. (2019) explored the survival rate of *Culex* mosquitoes under a series of constant and fluctuating temperature conditions and found that the survival rate was highest at a constant temperature of 25°C or a DTR with an average of 25°C. In this study, the survival rate of *Ae. albopictus* under DTR condition, whose average temperature was 27°C, is similar to that of 23°C and significantly higher than that of 31°C. The comparison of *Ae. albopictus* survival under a constant temperature of 27°C and a DTR condition with an average of 27°C would be performed in the future to explore how fluctuating temperature affects the longevity of mosquitoes.

At the high temperature, the number of eggs laid by *Ae. albopictus* was lower. The same result was found in *Anopheles, Culex*, and *Ae. aegypti* (Carrington et al., 2013a; Marinho et al., 2016; Abouzied, 2017), whose reproductive ability declined at high temperatures. In addition, it was found in *Culex paleus* that no eggs were oviposited by females at 32.5°C. Interestingly, a study about the oviposition of *Ae. albopictus* recorded no eggs laid at 20°C (Ezeakacha and Yee, 2019). The relationship between temperature and fecundity could be explained in two ways. First, mosquitoes need longer gonotrophic cycle lengths at low temperatures. Second, fecundity-size relationships suggest that females from lower larval rearing temperatures would be larger and more fecund, whereas those from higher larval rearing temperatures would be smaller and less fecund (Sibly, 1994; Garrad et al., 2016). It appears likely that the effect of larval rearing temperature could be carried over through female size to affect fecundity. In this experiment, the pupation rate and emergence rate of *Ae. albopictus* were the highest at 31°C, and the mosquito larvae growth duration was the longest at 23°C.

**Figure 5:**
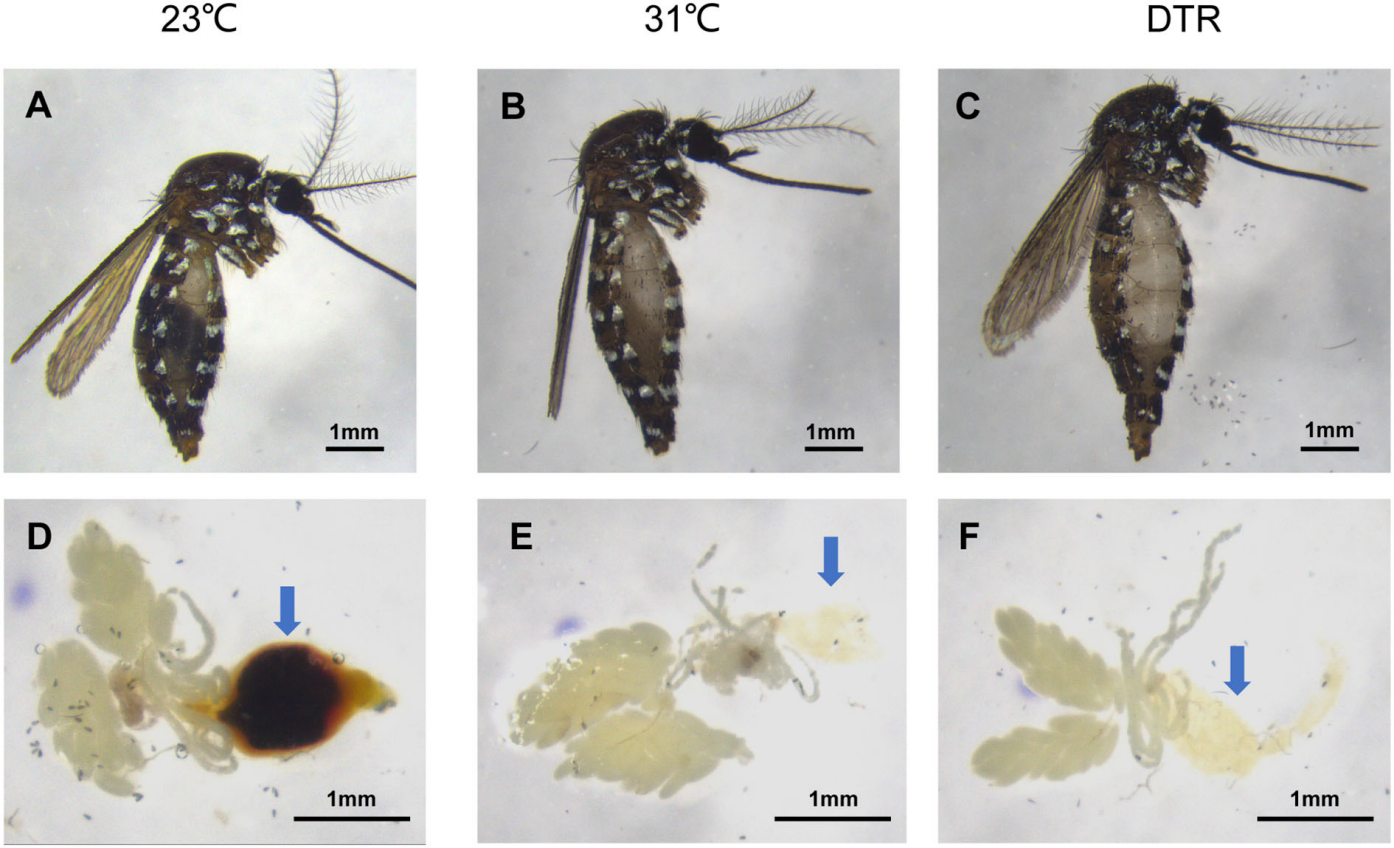
Digestion degree of blood bolus in *Ae. albopictus* infected with Zika virus at different temperatures 3dpi. Lateral view of the mosquito body **(A–C)**. Anatomical view of internal organs **(D–F)**. Arrow points to the midgut tissue.

It has been reported that increasing temperature lessens the mosquito gonotrophic cycle, thereby altering mosquito development and reproductive capacity (Afrane et al., 2006; Lardeux et al., 2008). This is due to the accelerated digestion of blood meal by mosquitoes at high temperatures, and mosquitoes obtained nutrients for rapid development and a series of reproductions (Sy et al., 2014). In this study, the mosquito midgut from each group was dissected on 3 dpi to examine blood digestion speed. It was found that the blood meal at 31°C and DTR had been basically digested and the blood meal in the midgut was invisible, while the blood meal in the midgut at 23°C was still visible (Figure 5), thus confirming this explanation.

For transovarial transmission, studies have confirmed that *Ae. aegypti* and *Ae. albopictus* can transmit the Zika virus through eggs (Li et al., 2017). In this study, we found that the IR at low temperature (23°C) was lower than that of 31°C and DTR. This is similar to the results of other studies on the effect of temperature on mosquito infection with viruses (Liu et al., 2017; Tesla et al., 2018; Blagrove et al., 2020). Low temperature reduces the ability of the virus to break through the mosquito midgut barrier, resulting in lower IRs and limiting virus invasion and propagation in mosquitoes. Under high temperatures, the IR of mosquitoes increases but so does the mortality rate, which also diminishes the vector competence of mosquitoes. In contrast, there are no significant differences in IR among those three temperature groups. Carrington et al. reported that DTR decreases IR but did not influence the transmission of DENV (Lambrechts et al., 2011; Carrington et al., 2013b,c).

### 4.2. Development time of larvae

The development time of *Ae. albopictus* is linearly related to temperature. Mosquito larvae take a longer time to develop into pupae at low temperatures, the development time required at high temperatures is significantly reduced compared to low temperatures, and similar results were found on the development time of *Culex* (Sibly, 1994; Afrane et al., 2006; Garrad et al., 2016). In this study, the development time of mosquitoes in the DTR was significantly shorter than the low-temperature group and longer than the high-temperature group. Richardson et al. found that fluctuating temperature had no effect on the development time of *Ae. aegypti* (Lardeux et al., 2008). However, there are also findings that the range of fluctuating temperatures is one of the factors affecting the development time of mosquitoes. When the temperature range of DTR is small (≤ 8°C), mosquito development time is not different between DTR and constant temperature, but when the temperature fluctuation range of the DTR group is 15°C, the large range of DTR will accelerate the development speed of mosquitoes (Sy et al., 2014).

Previous studies have shown that fluctuating temperatures are more accurate to investigate the effects of temperature on the mosquito life cycle (Bradshaw, 1980; Murdock et al., 2012a,b, 2013) because mosquitoes in the field live in fluctuating diurnal temperatures, not the constant temperature in the laboratory. While the results of this study showed that, compared to the constant temperature, DTR had complex effects on the biological parameters of mosquitoes. For example, in the DTR condition, the survival rate, number of eggs laid, hatching rate, pupation rate, and emergence rate of the *Ae. albopictus* infected with ZIKV were similar to the low-temperature group and different from the high-temperature group, while the development time and the viral load were similar to the high-temperature group and different from the low-temperature group. Our results implicated that not only temperature but also temperature pattern influence mosquito development, survival, and arbovirus transmission in a complicated way. Those findings may contribute to model construction that predicts vector-borne disease epidemics.

It should be noted that all conclusions of how temperature and temperature pattern influence the life cycle of *Ae. albopictus* in this study was based on the data from mosquitoes exposed to the Zika virus. Despite arboviruses generally not being highly pathogenic to mosquitoes (Patrican, 1985; Moncayo et al., 2000; Ciota et al., 2011), they sometimes alter mosquito biology depending on virus-mosquito species. For example, the chikungunya virus infection reduced the life span and shortened the time before the egg laying of *Ae. albopictus* Reunion strain but not the Mayotte strain (Martin et al., 2010). More interestingly, among the mosquitoes that were exposed to virus infection, those viral RNA-positive mosquitoes lived longer than that of virus negative mosquitoes (Ciota et al., 2011; Maciel-de-Freitas et al., 2011). It is possible that not only temperature pattern but also the interaction of temperature and virus infection contributed to differences in survival, fecundity, and development of *Ae. albopictus* between constant and fluctuating temperature conditions. However, our findings undoubtedly demonstrated that temperature significantly influences the survival of and virus transmission by mosquitoes under arbovirus infection status and suggest that vector competence evaluation should be conducted under fluctuating conditions for a more precise result.

## Data availability statement

The raw data supporting the conclusions of this article will be made available by the authors, without undue reservation.

## Author contributions

X-yJ: data curation. X-yJ, Y-tJ, X-xG, T-yZ, and J-hW: writing—review and editing. Y-tJ, T-yZ, J-hW, and X-xG: supervision. MW, NJ, and TC: perform infection experiments. X-xG, T-yZ, and J-hW: funding acquisition. All authors contributed to the article and approved the submitted version.
